# Synthesis and Transport Properties of ZnSnP_2-y_As_y_ Chalcopyrite Solid Solutions

**DOI:** 10.3390/ma17081712

**Published:** 2024-04-09

**Authors:** Daniel Ramirez, Luke T. Menezes, Holger Kleinke

**Affiliations:** Department of Chemistry and Waterloo Institute for Nanotechnology, University of Waterloo, Waterloo, ON N2L 3G1, Canada; dramirez@uwaterloo.ca (D.R.); ltmenezes@uwaterloo.ca (L.T.M.)

**Keywords:** chalcopyrites, pnictides, thermoelectric materials

## Abstract

This work focuses on the synthesis and properties of quaternary ZnSnP_2-y_As_y_ chalcopyrite solid solutions. Full miscibility of the solid solution is achieved using ball milling followed by hot press sintering. The measured electrical conductivity increases substantially with As substitution from 0.03 S cm^−1^ for ZnSnP_2_ to 10.3 S cm^−1^ for ZnSnAs_2_ at 715 K. Band gaps calculated from the activation energies show a steady decrease with increasing As concentration from 1.4 eV for ZnSnP_2_ to 0.7 eV for ZnSnAs_2_. The Seebeck coefficient decreases significantly with As substitution from nearly 1000 μV K^−1^ for ZnSnP_2_ to −100 μV K^−1^ for ZnSnAs_2_ at 650 K. Thermal conductivity is decreased for the solid solutions due to alloy phonon scattering, compared to the end members with y = 0 and y = 2, with the y = 0.5 and y = 1.0 samples exhibiting the lowest values of 1.4 W m^−1^ K^−1^ at 825 K. Figure of merit values are increased for the undoped solid solutions at lower temperatures when compared to the end members due to the decreased thermal conductivity, with the y = 0.5 sample reaching *zT* = 1.6 × 10^−3^ and y = 1 reaching 2.1 × 10^−3^ at 700 K. The largest values of the figure of merit *zT* for the undoped series was found for y = 2 with *zT* = 2.8 × 10^−3^ at 700 K due to the increasing *n*-type Seebeck coefficient. Boltztrap calculations reveal that *p*-doping could yield *zT* values above unity at 800 K in case of ZnSnAs_2_, comparable with ZnSnP_2_.

## 1. Introduction

Chalcopyrites *ABX*_2_ adopt an ordered supercell of the zincblende type, with a doubled *c* axis because of the ordering of the cations *A* and *B*. Typical representatives include charge-balanced pnictides *A*^+2^*B*^+4^(*Pn*^−3^)_2_ and chalcogenides *A*^+1^*B*^+3^(*Q*^−2^)_2_. The 2022 NIST Inorganic Crystal Structure Database lists 17 pnictides (*A* = Mg, Zn, and Cd; *B* = Si, Ge, and Sn; *Pn* = P, As, and Sb) and 26 chalcogenides (*A* = Li, Cu, and Ag; *B* = Fe, B, Al, Ga, In, and Tl; *Q* = S, Se, and Te) adopting the chalcopyrite type. For several decades, chalcopyrites have been studied for a number of different applications [1], including ZnGeP_2_ [2,3,4], AgGaSe_2_ [5] and others [6] as nonlinear optical materials, photovoltaics [7], LEDs [8] and thermoelectric materials [9,10].

Thermoelectric (TE) materials are capable of converting the abundant otherwise lost waste heat into useful electricity, which may contribute to more sustainable energy generation [11,12,13,14]. Additionally, today’s researchers focus also on investigations into powering the countless sensors in the Internet of Things [15,16,17], and utilizing body heat for various sensors [18,19,20,21], both with TE materials. TE materials are classified by their figure of merit, *zT*, with classical materials having peak values around unity. Recent progress in, for example, nanostructuring and utilizing the phonon glass electron crystal (PGEC) approach [22] to lower thermal conductivity, led to significantly higher values, even exceeding *zT*_max_ = 2 at high temperatures in selected examples such as PbTe with nanodomains [23] or with SrTe nanoadditions [24], nanostructured Cu_2_Se [25] and Cu_2_Se/CuInSe_2_ nanocomposites [26], and *p*-doped SnSe [27,28,29].

For the most part, phosphides and arsenides are neither among the best performing thermoelectrics nor among the heavily investigated ones, mostly because they typically comprise higher thermal conductivity than the more traditional antimonides and tellurides. Notable exceptions exist however [30,31,32], with figure of merit values of the order of 1 for both *p*- and *n*-doped phosphides [33]. Several Zn-based chalcopyrite phosphides and arsenides (Zn*BPn*_2_ with *B* = Si, Ge, and Sn; *Pn* = P and As) were predicted to have high thermopower [34]. We recently experimentally demonstrated that despite the high symmetry crystal structures and relatively light constituent elements, the solid solutions ZnGe_1-x_Sn_x_P_2_ can achieve reasonably low thermal conductivity, and ultimately high figure of merit values when properly doped [35]. Here, we report on the solid solutions ZnSnP_2-y_As_y_, focusing on Sn instead of Ge because of its lower price, higher abundancy, and (partially) substituting As for P because of its higher weight despite its higher toxicity, as higher weight typically occurs with lower lattice thermal conductivity [13]. 

## 2. Materials and Methods

All reactions began from the elements (Zn powder (99.9% Alfa Aesar, Tewksbury, MA, USA, −100 mesh), Sn powder (99.998% Alfa Aesar, −100 mesh), Ge pieces (99.999% STREM Chemicals, Newburyport, MA, USA), P powder (99% Alfa Aesar, −100 mesh), and As powder (99.98% Alfa Aesar, −100 mesh), which were loaded into zirconia lined ball mill jars with ~10 g of 1 mm zirconia balls in an argon filled glove box. The jars were milled at 600 rpm for 5 min increments with 1 min rest times, with the direction reversed after each rest time, using the Fritsch Pulverisette 7, (acquired from Laval Lab, Laval, QC, Canada). Three milling steps were employed, all as described above; after the first milling step of 5 h, the jars were opened in the argon glovebox and agglomerated materials were mechanically reincorporated before the two final 2 h milling steps. After those steps, the reacted materials were ground by hand to yield a uniform micro-crystalline sample. Finally, high-pressure sintering was performed as a final reaction step in graphite dies of a diameter of 12.7 mm under a pressure of 56 MPa using an Oxy-Gon Industries (Epsom, NH, USA) hot press; the temperature was ramped up over two hours to at 850 K and held there for 6 h, followed by a pressure-free cooldown.

Powder X-ray diffraction (PXRD) was performed on the ground samples as well as polished pellets at room temperature using the Inel (Artenay, France) powder X-ray diffractometer, which utilizes a position sensitive detector and Cu Kα_1_ radiation. Rietveld refinements were performed using the GSAS-II (v. 5761) analysis software [36].

An FEI (Hillsboro, OR, USA) Quanta FEG ESEM microscope was used for the energy-dispersive analysis of X-ray (EDAX) measurements with an acceleration voltage of 20 kV. Five-point measurements were taken for each sample and then averaged, and area scans and elemental mapping were performed on a 150 μm × 150 μm area. 

Thermal diffusivity was measured on the pressed pellets under argon using the TA Instruments (Hillsboro, OR, USA) DLF-1200 system. The Seebeck coefficient was measured by the direct method, and electrical conductivity measured by a standard 4-point method, both with the ULVAC RIKO ZEM-3 apparatus under helium on rectangular pellets of 8 × 2 × 2 mm, cut from the original round pellet after the thermal diffusivity measurements. Estimated measurement errors are 3% for the Seebeck coefficient, 5% for the electrical conductivity [37], and 5% for the thermal conductivity measurements [38], resulting in 10% for the figure of merit. The error bars were included in the corresponding figures. 

Electronic structure calculations were carried out using the WIEN2k_21.1 package that employs the full potential linearized augmented plane wave (LAPW) method [39,40,41]. Here, we used the generalized gradient approximation (GGA) from Perdew, Burke and Ernzerhof (PBE) [42]. We used a grid of 1000 *k* points evenly distributed throughout the first Brillouin zone, which resulted in 99 symmetry independent *k* points for ZnSnAs_2_. As convergence criterion, we used 10^−4^ Ry for the maximum energy change. In addition, we employed the BoltzTraP2 (v22.3.1) package [43] that uses the Boltzmann transport equations to calculate the thermoelectric properties. Although assuming a constant relaxation time results in an additional uncertainty [44,45], this often leads to good agreement between theory and experiment [46]. 

## 3. Results and Discussion

### 3.1. Chemical and Structural Characterization

**Figure 1:**
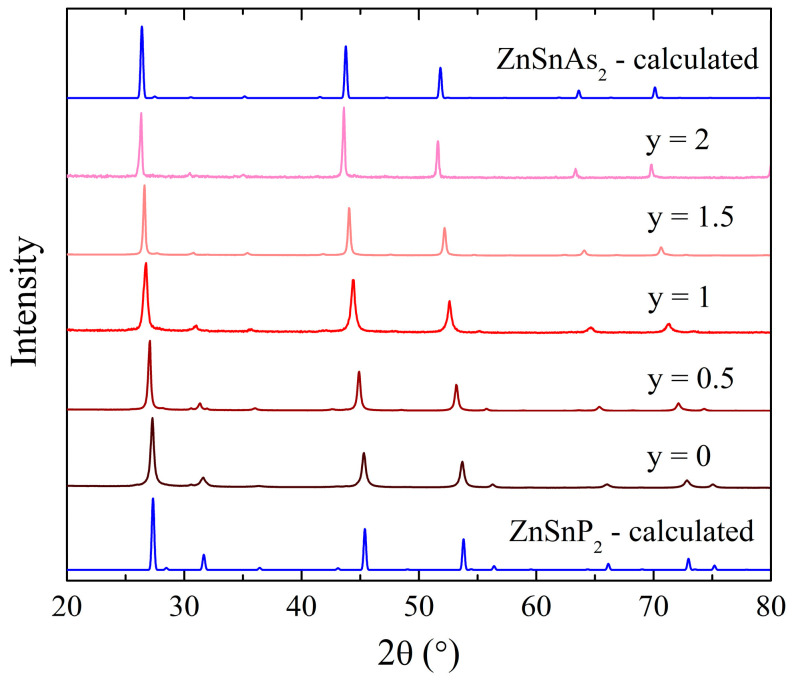
PXRD patterns of ZnSnP_2-y_As_y_ series (y = 0, 0.5, 1, 1.5, 2) with calculated patterns for comparison (ZnSnP_2_: ICSD 22179; ZnSnAs_2_: ICSD 611439).

After thermal diffusivity measurements were performed, the samples were cut into bars for transport measurements, and PXRD was performed on the leftover pellet pieces hand ground into powders. Long time (>12 h) PXRD measurements for ZnSnP_2-y_As_y_ were performed for y = 0, 0.5, 1, 1.5, and 2 (Figure 1). The patterns are very consistent and do not exhibit any signs of any side products. 

**Figure 2:**
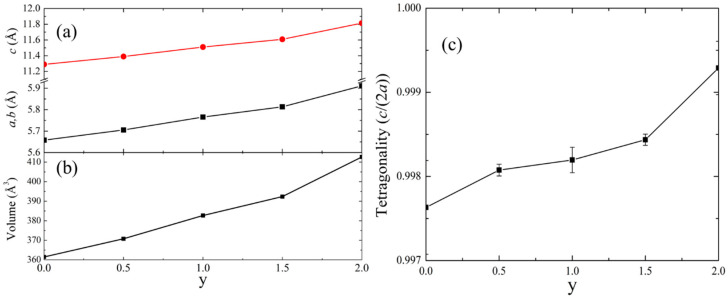
Rietveld refinement results for ZnSnP_2-y_As_y_. (**a**) unit cell parameters; (**b**) volume; (**c**) tetragonality.

A shift of the characteristic peaks to lower angles occurs with increasing As concentration due to the unit cell expansion, caused by the larger size of the As atoms, compared to the P atoms. The expansion of unit cell parameters is illustrated in Figure 2a,b, and summarized in Table 1, namely a relatively steady increase with increasing As concentration from y = 0 to y = 1.5, followed by a larger increase when moving from y = 1.5 to y = 2. The tetragonality of the system, defined as *c*/(2*a*), slowly increases from 0.998 at y = 0 to 1.000 at y = 2 (Figure 2c). 

The chemical compositions of the series were evaluated by refining the occupancy parameters during Rietveld refinements as well energy dispersive X-ray spectroscopy (EDAX) analysis. The y values (As content) of the solid solutions were refined to 0.59(1), 1.06(3), and 1.66(1) for the solid solutions with nominal y values of 0.5, 1.0, and 1.5. EDAX results for the solid solutions and end members can be found in Table 2. The concentrations determined from EDAX measurements match well for the solid solutions with expected atomic percent values showing differences of less than 8%, while the end member ZnSnAs_2_ displayed significantly lower than expected Zn and As (higher Sn) concentration. EDAX atomic mapping for these materials can be found in the Supplementary Information (Figure S1). 

### 3.2. Experimental Physical Properties

**Figure 3:**
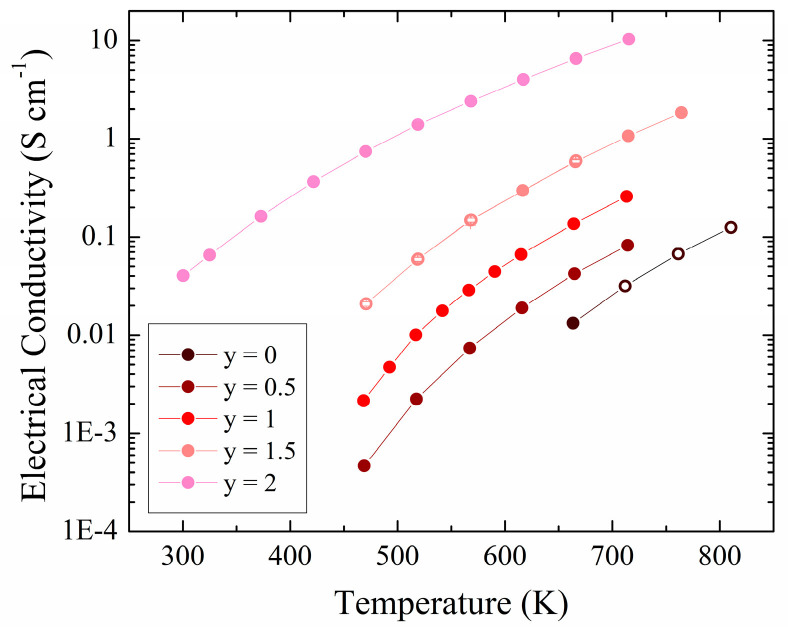
Electrical conductivity for ZnSnP_2-y_As_y_. Open circles: data collected during cooldown.

Electrical conductivity versus temperature measurements were carried out for all members of the series (Figure 3). We verified the stability of the samples under the measurement conditions by measuring a few additional datapoints during cooldown. These datapoints (open circles in Figure 3) match the corresponding points obtained during heating to approximately 800 K very nicely, confirming the samples’ stabilities. The electrical conductivity of the P-rich members was too low to be measured at room temperature, but became measurable around 450 K or above, depending on y. As expected for semiconductors, the electrical conductivity increases with temperature for all members. An overall increase in conductivity with increasing As concentration is evident, in line with the expected decreasing band gap and higher degree of covalency (which increases carrier mobility), both caused by the lower electronegativity of As, compared to P. The conductivity for ZnSnP_2_ rises from *σ* = 0.01 S cm^−1^ at 650 K to 0.1 S cm^−1^ at 800 K, and for ZnSnAs_2_ from *σ* = 0.03 S cm^−1^ at 300 K to 7.0 S cm^−1^ at 650 K. 

For comparison, ZnSnAs_2_ crystals grown by chemical vapor transport displayed a *σ* value of only 1.4 × 10^−^^4^ S cm^−1^ at 300 K, much lower than observed in this work, likely due to the low level of impurities typically found in perfect single crystals [47]. On the other hand, single crystals synthesized by the Bridgman method displayed *σ* = 24 S cm^−1^ at 295 K [48]. ZnSnAs_2_ bulk samples studied under various heat treatments had *σ* values ranging from 0.1 S cm^−1^ to 1200 S cm^−1^ at 300 K, with most values around 400 S cm^−1^ [49]. Heat treatments of the quenched samples tended to decrease conductivity, indicating healing of possible defects in these samples, which then resulted in lower electrical conductivity. Chalcopyrite CuInSe_2_ single crystals exhibited σ values ranging from 0.15 S cm^−1^ at 300 K to 0.46 S cm^−1^ at 575 K [50], likely low because of their low amounts of intrinsic defects. Studies of vacancy doped *p*-type Cu_0.99_InSe_2.05_—which adopts the same structure type and has the same number of electrons—displayed σ values ranging from 1.5 S cm^−1^ at 325 K to 6.0 S cm^−1^ at 760 K with degenerate semiconducting behavior [51]. 

**Figure 4:**
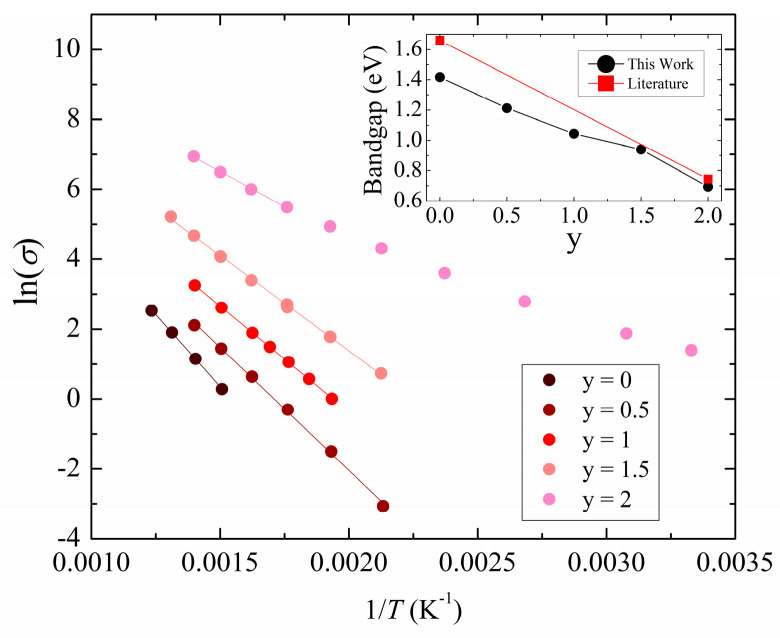
Arrhenius plots of the electrical conductivity for ZnSnP_2-y_As_y_.

An Arrhenius plot of ln(*σ*) versus *T^−^*^1^ is shown in Figure 4, where a linear trend is expected for intrinsic semiconductors and curved trends for extrinsic semiconductors. The series members displayed mostly intrinsic semiconducting behavior, except for ZnSnAs_2_ with its extrinsic behavior indicative of a significant amount of defects. Band gaps of the series members are calculated from the slope (in the high temperature linear region) using the Arrhenius expression for the activation energy. A clear trend of decreasing band gaps with As concentration is observed as postulated above (inset of Figure 4). The previously determined band gaps of *E*_g_ = 1.4 eV for ZnSnP_2_ and 0.7 eV for ZnSnAs_2_ are comparable to the literature values of 1.68 eV for ZnSnP_2_ [52] and 0.59 eV for ZnSnAs_2_ [48]. 

**Figure 5:**
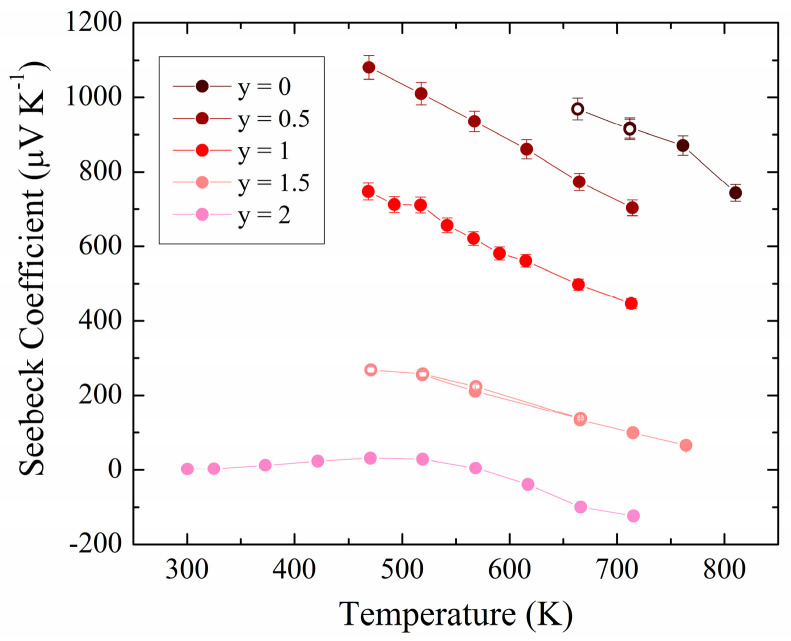
Seebeck coefficient for ZnSnP_2-y_As_y_. Open circles: data collected during cooldown.

The Seebeck coefficient data measured for the full series are displayed in Figure 5. The values decrease with increasing As concentration, for example at the highest temperatures from *S* = 744 μV K^−1^ for y = 0 down to −125 μV K^−1^ for y = 2, in line with the opposing trend in the electrical conductivity. The temperature dependence for the materials from y = 0 to y = 1.5, decreasing steadily with increasing temperature, is typical of *p*-type intrinsic behavior. A turnover of the slope in Seebeck versus temperature is indicative of bipolar conduction, which is seen at 475 K for the y = 2 end member, which ultimately results in a *p*-type to *n*-type transition. 

Previous studies of bulk ZnSnAs_2_ showed degenerate *p*-type semiconducting behavior, with Seebeck coefficient values ranging from *S* = 41 μV K^−1^ at 300 K to 60 μV K^−1^ at 440 K similar to the results found in this work for the same temperature range. Various heat treatments and synthesis methods produced room temperature *S* values ranging from 26 μV K^−1^ (slowly cooled sample) to 224 μV K^−1^ (after annealing at 883 K) [49]. Single crystals displayed larger overall S values with degenerate semiconducting behavior, ranging from 310 μV K^−1^ at 300 K to 400 μV K^−1^ at 600 K [48]. The analogous I–III–VI chalcopyrite CuInSe_2_ single crystals grown by vapor deposition displayed *S* values ranging from 542 μV K^−1^ at 300 K down to 300 μV K^−1^ at 400 K, and then increasing again to 600 μV K^−1^ at 625 K [50]. Vacancy doped *p*-type Cu_0.99_InSe_2.05_ had *S* values of 300 μV K^−1^ at 300 K, which increased to 625 μV K^−1^ at 620 K to finally decrease to 500 μV K^−1^ at 775 K [51]. A similar p-type to *n*-type transition was observed in one study of CuInSe_2_, with 100 μV K^−1^ at 300 K increasing slightly to 200 μV K^−1^ at 390 K and then decreasing rapidly to −200 μV K^−1^ at 560 K [53]. 

**Figure 6:**
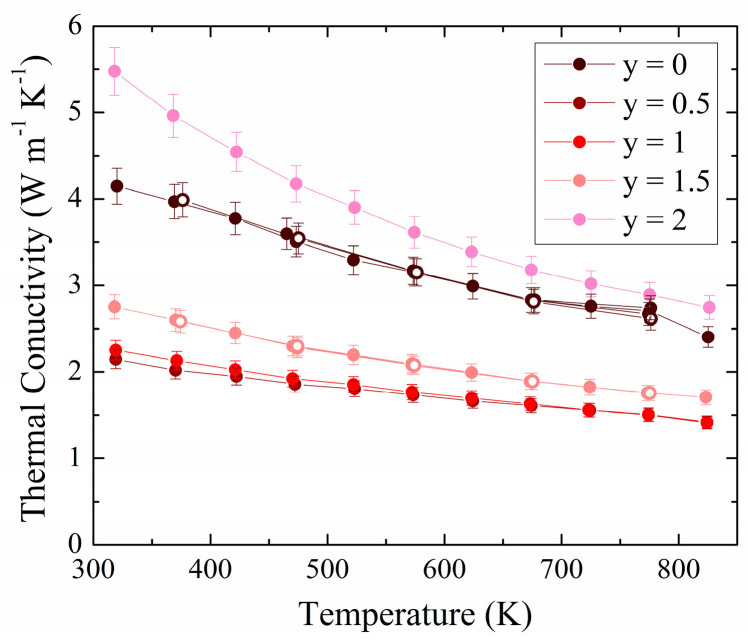
Thermal conductivity for ZnSnP_2-y_As_y_. Open circles: data collected during cooldown.

The thermal conductivity data are shown in Figure 6, revealing a typical decrease with temperature due to increased phonon frequencies. Here as well, the data collected after the first heating cycle during the cooldown (open circles) match the data during heating, showing stability of the materials under the measurement conditions. The end members exhibit the largest values with *κ* = 4.2 W m^−1^ K^−1^ and 5.6 W m^−1^ K^−1^ at 300 K to 2.4 W m^−1^ K^−1^ and 2.7 W m^−1^ K^−1^ at 825 K for y = 0 and y = 2, respectively. The lowest values are observed in y = 0.5 and y = 1 with *κ* = 2.2 W m^−1^ K^−1^ and 2.3 W m^−1^ K^−1^ at 300 K, respectively, which have equal values of 1.4 W m^−1^ K^−1^ at 825 K. Slightly larger than the other solid solutions, the material with y = 1.5 displays *κ* values between 2.8 W m^−1^ K^−1^ at 300 K and 1.7 W m^−1^ K^−1^ at 825 K. Increased phonon scattering due to mass fluctuation effects and disorders thus reduces the thermal conductivity by more than a factor of two at room temperature for the solid solutions compared to the end members. 

Considering the low electrical conductivity values, the electronic contribution to the measured total thermal conductivity remains always below 0.01 W m^−1^ K^−1^ based on the Wiedemann-Franz law. Therefore, the measured thermal conductivity is basically directly equal to the lattice thermal conductivity. Gasson et al. measured between *κ* = 4.3 W m^−1^ K^−1^ (sintered sample) and 14.1 W m^−1^ K^−1^ (slowly cooled sample) for ZnSnAs_2_ [49], demonstrating again how the properties, e.g., charge carrier concentration and thus electronic thermal conductivity, depend on the heat treatment. 

**Figure 7:**
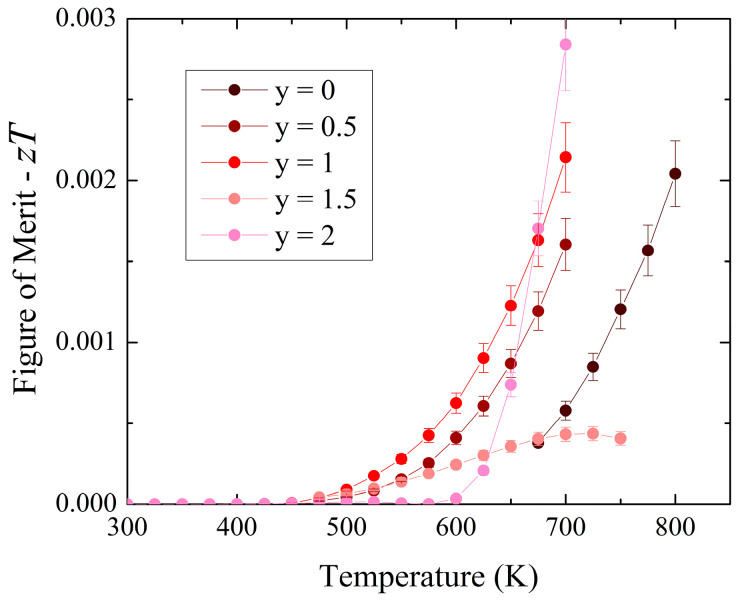
Thermoelectric figure of merit for ZnSnP_2-y_As_y_.

The calculated thermoelectric figure of merit is obtained from combining Seebeck coefficient, electrical and thermal conductivity, as well as temperature via *zT* = *TσS*^2^ *κ*^−1^. As the thermal data were obtained for a larger temperature range, the *zT* values are limited to the temperature ranges of the electrical measurements. The largest and smallest *zT* values are observed in the y = 2 end member with *zT* = 1.4 × 10^−^^9^ at 300 K to 0.003 at 700 K and hypothetically zero at the *p*-*n* transition temperature (Figure 7). At high temperatures, the relatively large electrical conductivity, increasing negative Seebeck coefficient, and decreasing thermal conductivity contribute to improving thermoelectric performance. Gasson et al. determined *zT* of slowly cooled ZnSnAs_2_ to be 0.004 at 300 K, with a carrier concentration of 5.7 × 10^20^ cm^−1^ [49]. The maximum *zT* values for all solid solution members (i.e., containing both P and As) occur at 700 K with 2.0 × 10^−^^3^ for y = 1, 1.6 × 10^−^^3^ for y = 0.5, and 4.3 × 10^−^^4^ for y = 1.5. The ZnSnP_2-y_As_y_ solid solutions outperform the y = 0 end member. 

### 3.3. Calculated Physical Properties

**Figure 8:**
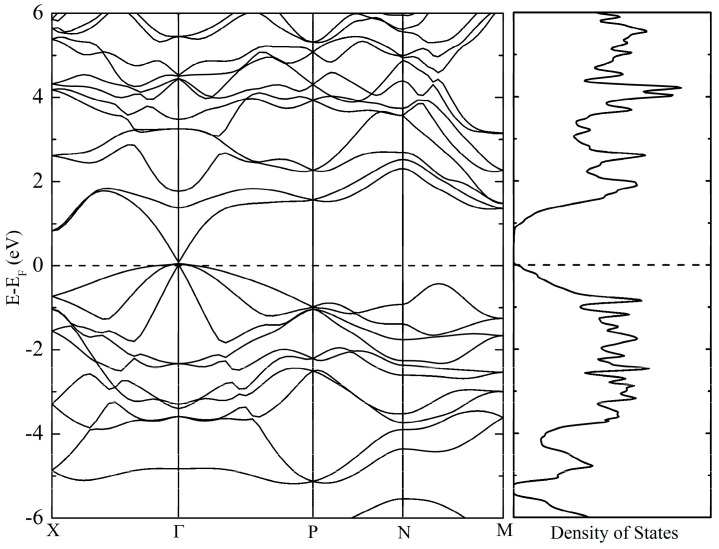
Band structure (left) and figure of merit (right) for ZnSnAs_2_.

The band structure and density of states of ZnSnAs_2_ are shown in Figure 8, as the corresponding results for ZnSnP_2_ were published before [35]. A small direct band gap exists at the Γ point, with several bands converging at the top of the valence band, in line with the high degree of tetragonality. There are strong resemblances to the calculated band structure of ZnSnP_2_ [35], with the smaller band gap being the most noticeable difference. 

**Figure 9:**
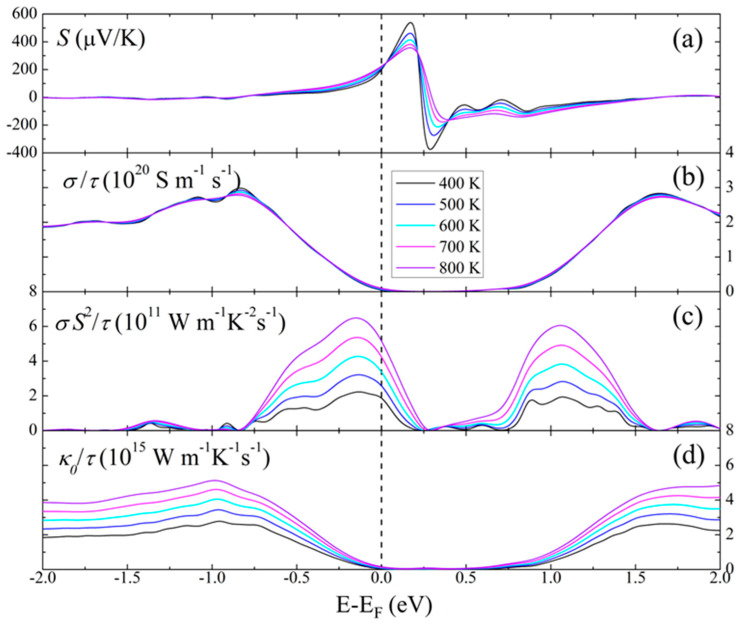
BolzTraP2 calculation results for ZnSnAs_2_: (**a**) Seebeck coefficient *S*; (**b**) electrical conductivity *σ*, (**c**) power factor *σS*^2^, and (**d**) electronic thermal conductivity *κ*_0_.

Using BoltzTraP2, the Seebeck coefficient S, the electrical conductivity σ, the power factor σS^2^, and the electronic contribution to the thermal conductivity *κ*_0_ were calculated (relative to the relaxation time τ) for temperatures up to 800 K, as displayed in Figure 9. Depending on the carrier concentration (relative energies), high Seebeck values around +500 μV K^−1^ and −400 μV K^−1^ may be achievable at 400 K, and power factor values of S^2^σ τ^−1^ = 6 × 10^11^ W m^−1^ K^−^^2^ s^−1^ for both *p*- and *n*-type at 800 K. For comparison, the corresponding peak values were 13 × 10^11^ W m^−1^ K^−^^2^ s^−1^ for ZnGeP_2_ and 11 × 10^11^ W m^−1^ K^−^^2^ s^−1^ for ZnSnP_2_ (both at 900 K). 

To obtain estimated figure of merit values for ZnSnAs_2_, we used a standard relaxation time of *τ* = 10 fs, as typical for these materials [54], and the experimentally obtained lattice thermal conductivity values *κ_lat_* (Equation (1)):

(1) $$zT=T\frac{(\sigma S^{2}/\tau )\cdot \tau }{(\kappa_{0}/\tau )\cdot \tau +\kappa_{lat}}$$

**Figure 10:**
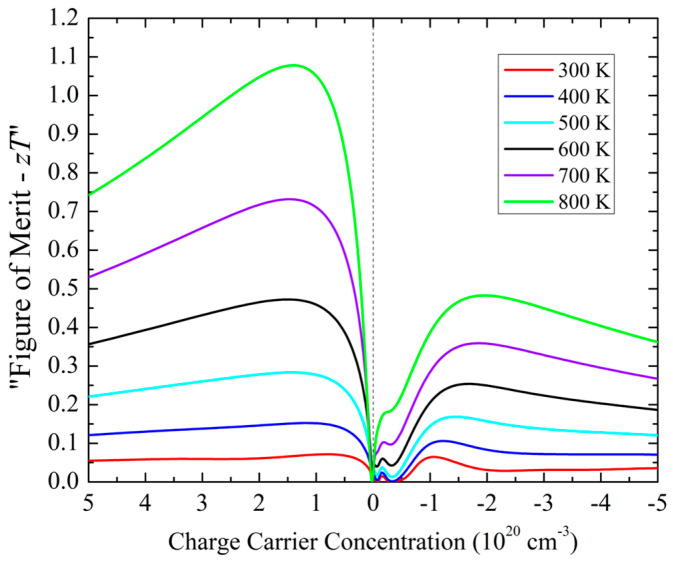
Estimated figure of merit for ZnSnAs_2_.

As shown in Figure 10, the *zT* values increase steadily from room temperature up to 800 K, reaching values slightly above unity (*zT* = 1.07, at 1.4 × 10^20^ carriers per cm^3^, or 0.006 carriers per formula unit) for p-type and 0.48 for *n*-type doped ZnSnAs_2_ at 1.4 × 10^20^ carriers per cm^3^. The maximum for the *p*-type compares well with ZnSnP_2_, where a *zT*_max_ = 1.0 was obtained at 800 K. 

While the work from Gasson et al. implies that a large range of different charge carrier concentrations of between 0.2 × 10^20^ cm^−1^ and 33 × 10^20^ cm^−1^ can be obtained for ZnSnAs_2_ [49], a systematic doping study has not yet been performed on ZnSnAs_2_. *p*-type doping could be systematically achieved by partial replacements of Zn with Cu or Sn with In, with formulae of Cu_0.006_Zn_0.994_SnAs_2_ and ZnIn_0.006_Sn_0.994_As_2_, respectively, corresponding to a hole carrier concentration of the order of 10^20^ cm^−^^3^. 

## 4. Conclusions

We successfully synthesized and characterized the solid solution series ZnSnP_2-y_As_y_ via a mechanochemical route. Full miscibility exists, and the increasing As concentration causes a smaller band gap and higher electrical conductivity. The lowest thermal conductivity values were measured for the solutions with y = 0.5 and y = 1.0. 

The undoped as-prepared samples all exhibit poor thermoelectric performance (low figure of merit). Our calculations showed that proper *p*-type doping of ZnSnAs_2_ should lead to outstanding performance with figure of merit values exceeding *zT* = 1, while *n*-doping would be less successful with peak *zT* values of the order of 0.5. As previously demonstrated [35], the other end member, ZnSnP_2_, should be able to achieve comparable performance when *p*-doped, and better performance when *n*-doped. 

## Figures and Tables

**Table 1:** Rietveld refinement results for ZnSnP_2-y_As_y_.

y	*a*, *b* (Å)	*c* (Å)	*V* (Å^3^)	*c*/(2*a*)
0.0	5.6584(14)	11.290(4)	361.48(30)	0.9976(5)
0.5	5.7057(2)	11.3895(5)	370.79(1)	0.9981(1)
1.0	5.7659(4)	11.511(1)	382.69(3)	0.9982(2)
1.5	5.8133(2)	11.6085(5)	392.31(1)	0.9984(1)
2.0	5.9112(8)	11.814(2)	412.81(5)	0.9993(3)

**Table 2:** EDAX analysis results in atomic-% obtained using five-point measurements for ZnSnP_2-y_As_y_.

Element	y = 0.5	y = 1.0	y = 1.5	y = 2.0
Zn	24.8	23.9	24.8	21.9
Sn	25.8	24.8	25.6	32.1
P	37.5	27.1	12.7	-
As	11.9	24.2	36.9	45.9

## Data Availability

Data are contained within the article and Supplementary Materials.

## Supplementary Materials

The following supporting information can be downloaded at: https://www.mdpi.com/article/10.3390/ma17081712/s1, Figure S1: EDAX atomic mapping.
