# Supplementary material for: Synthesis and Transport Properties of ZnSnP2-yAsy Chalcopyrite Solid Solutions
Source: Materials (Basel). 2024 Apr 9;17(8):1712. doi: 10.3390/ma17081712 (PMC11050980; doi:10.3390/ma17081712)
Supplement: Supplementary file 1 [file materials-17-01712-s001.zip › materials-2920988-supplementary.pdf]

Synthesis and Transport Properties of $ZnSnP_{2-y}As_y$ Chalcopyrite Solid Solutions

Daniel Ramirez [1], Luke T. Menezes [1] and Holger Kleinke [1,*]

[1] Department of Chemistry and Waterloo Institute for Nanotechnology, University of Waterloo
* Correspondence: kleinke@uwaterloo.ca

Supplementary information

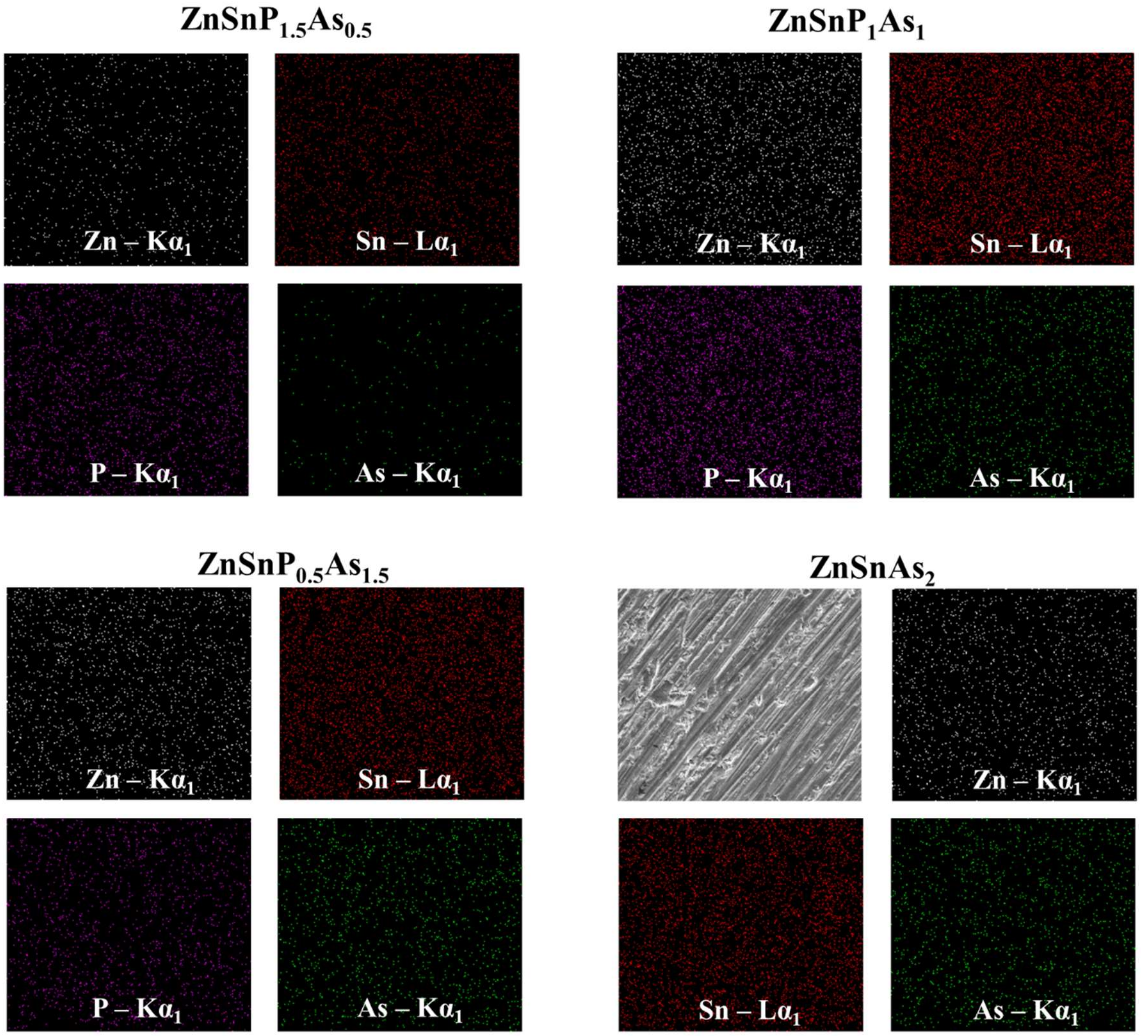

Figure S1: EDAX atomic mapping.
